# Aromatase inhibitors and risk of cardiovascular events in breast cancer patients: a systematic review and meta-analysis

**DOI:** 10.1186/s40360-019-0339-1

**Published:** 2019-10-29

**Authors:** Yang He, Jianhua Zhang, Guofang Shen, Lin Liu, Qingwei Zhao, Xiaoyang Lu, Hongyu Yang, Dongsheng Hong

**Affiliations:** 1 Department of Pharmacy, the First Affiliated Hospital, College of Medicine, Zhejiang University, 79 Qingchun Road, Hangzhou, 310003 People’s Republic of China; 2 College of Medicine, Zhejiang University, 866 Yuhangtang Road, Hangzhou, 310003 People’s Republic of China; 3 Department of Management, the Logistics Service Center of Municipal Government, Hangzhou, 310019 People’s Republic of China; 4 Loma Linda University School of Pharmacy, Loma Linda, CA 92354 USA

**Keywords:** Breast cancer, Aromatase inhibitors, Cardiovascular events, Meta-analysis

## Abstract

**Background:**

Cardiovascular events (CVEs) was considered as one of the primary cause to reduce the quality of life in breast cancer patients with aromatase inhibitors (AIs) treatment, which has not been sufficiently addressed. The aim of this study was to assess the correlation between risk of CVEs and AIs in patients with breast cancer.

**Methods:**

Included studies were obtained from the databases of Embase, Pubmed, Cochrane Library, Clinical Trials.gov, and reference lists. The main outcome measures were overall incidence, odds ratios (ORs), and 95% confidence intervals (CIs). Furthermore, the association and the risk differences among different tumor types, AIs,ages,or treatment regimens were conducted. Fixed-effect or random-effect models were applied in the statistical analyses according to the heterogeneity. Our analysis was performed according to the Preferred Reporting Items for Systematic Reviews and Meta-Analyses (PRISMA) statement.

**Results:**

Seventeen studies, which included 44,411 subjects, were included in our analyses. The overall incidence of CVEs in AIs group was 13.02% (95% CI: 8.15–20.17%) and almost all of the high-grade CVEs occurred in patients treated with AIs. The pooled ORs of CVEs was 0.9940 (95% CI: 0.8545–1.1562). Under sub-group analysis, the incidence of CVEs related to exemestane was higher than that of controls (OR = 1.1564, 95% CI: 1.0656–1.2549), but no statistical differences in risk of CVEs were found in other sub-group analysis. No evidence of publication bias was found for incidence of CVEs in our meta-analysis by a funnel plot.

**Conclusions:**

These results suggest that patients with breast cancer treated with AIs do not have a significant risk of developing CVEs in comparison with the controls, and exemestane might not be considered as the alternative AI to the breast cancer patients from the perspective of CVEs. Further studies are recommended to investigate this association and the risk differences among different tumor types, AIs or treatment regimens.

## Background

Breast cancer is an increasing public health problem throughout the world, which is one of the most common malignancies and causes for tumor-related deaths among women [1–3]. Fortunately, The 5-year survival rate for patients with breast cancer has elevated from an average of 53% in 2007 to 85% in 2012 and the number is still rising in recent year [4, 5]. However, this positive trend in improved cancer-related mortality is weakened by an emerging increase in cardiovascular (CV) morbidity and mortality in these patients. It is estimated that over $800 million will be spent annually in the US on providing CV care for these women with breast cancer [2, 6]. Several recently published research studies have studied possible etiologies of these events, and it is suggested that the increased morbidity of CV disease and events has a temporal relationship with the administration of chemotherapy for cancer [7–11]. Nevertheless, the causes for this increase in cardiovascular related events has not been clearly demonstrated.

Anastrozole, letrozole, and exemestane are the three proven aromatase inhibitors (AIs), and several large randomized control trials (RCTs) have demonstrated the advantage of the AIs compared with tamoxifen [12–14].AIs have systematically been clarified to increase the incidence of genitourinary and musculoskeletal discomfort compared to the control group [15, 16], and of symptoms related to fractures [17]. However, the potential impact on cardiovascular system has not been sufficiently elucidated, and patients with cardiovascular disease risk is difficult to identify. Cardiovascular events (CVEs) related to breast cancer include hypertension, ischemic cardiovascular disease, venous thrombosis, hypercholesterolaemia, arrhythmia, cardiac failure, peripheral arterial disease, embolism, myocardial infarction, atrial fibrillation [18–21]. Data regarding the CVEs in AIs treated patients was reported in several RCTs, several of these reporting increased risks with AIs [7, 22–24]. However, recent study suggested that AIs may not increase the risk of the most fatal cardiovascular events [9]. In consideration of CVEs could greatly reduce the quality of life in breast cancer patients. Thus it is of great importance to fully understand the incidence of CVEs related to AIs treatment. Therefore, we conducted a systematic review and meta-analysis to evaluate the association of AI therapies with CVEs.

## Methods

### Search strategy

A systemic search was conducted on PubMed (from 1967), Embase (from 1974), and the Cochrane Library electronic databases at the end of December 2017. The keywords ‘Aromatase inhibitors’, ‘Breast cancer’, ‘Randomized controlled trials’, ‘Clinical trials’ and ‘Controlled clinical trials’ were used for the search. Moreover, we also searched for registered clinical trials on ClinicalTrials.gov. Only clinical trials and articles published in English were included in this study. This study is an meta-analysis and not involves subjects, ethical approval was not required.

### Study selection and quality assessment

All studies were assessed by two review investigators (HY and ZJH)independently. Trials which were judged as pertinent by one of the investigators were retrieved for further consideration. All identified discrepancies were identified and resolved by consensus. Clinical trials were included in the current study if they met the following criteria:

- The trial involved patients diagnosed with breast cancer.
- The trial was prospective phase II or III RCTs and involved subjects receiving AIs treatment.
- The data for events of CVEs was available in the trial.

The Jadad scale was used to assess the quality of each included trail. A higher score in the range of 0 to 5 indicated a high quality [25].

### Data extraction and clinical endpoints

Two investigators (HY and ZJH) performed data extraction independently. The following information was extracted from each trial: first author’s name, year of publication, trials phase, ethnicity, number of patients in the AIs and control groups, CVEs. These clinical end points were obtained according to the Common Terminology Criteria for Adverse Events (CTCAE) of National Cancer Institute (https://ctep.cancer.gov/protocolDevelopment/electronic_applications/ctc.htm), and a variety of CVEs, such as hypertension, ischemic cardiovascular disease, venous thrombosis, hypercholesterolaemia, arrhythmia, cardiacfailure, peripheral arterial disease, embolism, myocardial infarction and atrial fibrillation were included. A composite of all-cause mortality and cardiac events, such as non-fatal myocardial infarction, new atrial fibrillation or heart failure episode requiring hospitalization was used as a primary end point in the risk assessment of CVEs, and secondary end points comprised the primary end point composite factors. The primary end point was defined as the time from cancer diagnosis to the first occurrence of any component of the composite major adverse cardiovascular event outcome.

### Data analysis

The data analysis was carried out in accordance with the Preferred Reporting Items for Systematic Reviews and Meta-Analyses (PRISMA) statement [26, 27] The major indexes were incidence, odds ratio (OR), and corresponding 95% CIs of relevant CVEs. CVEs Subjects and the total number of subjects in groups with AIs treatment were extracted from the safety profiles of included trials to calculate CVEs incidence. The confidence level sets the boundaries of a confidence interval (CI), and it is conventionally set at 95% to coincide with the 5% convention of statistical significance in hypothesis testing. A 95% CI is the interval that you are 95% certain contains the true population value as it might be estimated from a much larger study. From each trial, we derived the proportion and 95% CI of patients developed CVEs. For trials with a control group, we further derived the OR of CVEs. For trials reporting no CVEs in any group, the OR and variance was obtained using classic half-integer continuity correction. The data was tested for heterogeneity and among-study inconsistency using the Cochrane’s Q statistic and I^2^ tests respectively [28, 29]. The statistical significance of heterogeneity was marked by a *P* < 0.1 or I^2^ > 40%. The random-effects model was applied for data analysis when heterogeneity existed. Otherwise, a fixed-effects model was selected. A *p*-value<0.05 was considered statistically significant. The publication bias was estimated by the Begg’s and Egger’s test and a contour-enhanced funnel plots was conducted to futher evaluate the publication bias and enhance interpretation of a funnel plot by helping distinguish publication bias from other cause of funnel plot asymmetry [30–32]. Meta-Analyses (PRISMA) Statement was shown in the Additional file 5: Table S1. All data analyses were performed by using R software, version 3.2.3 (The R foundation for statistical computing, http://www.r-project.org) .

## Results

### Search results and trial characteristics

**Fig. 1:**
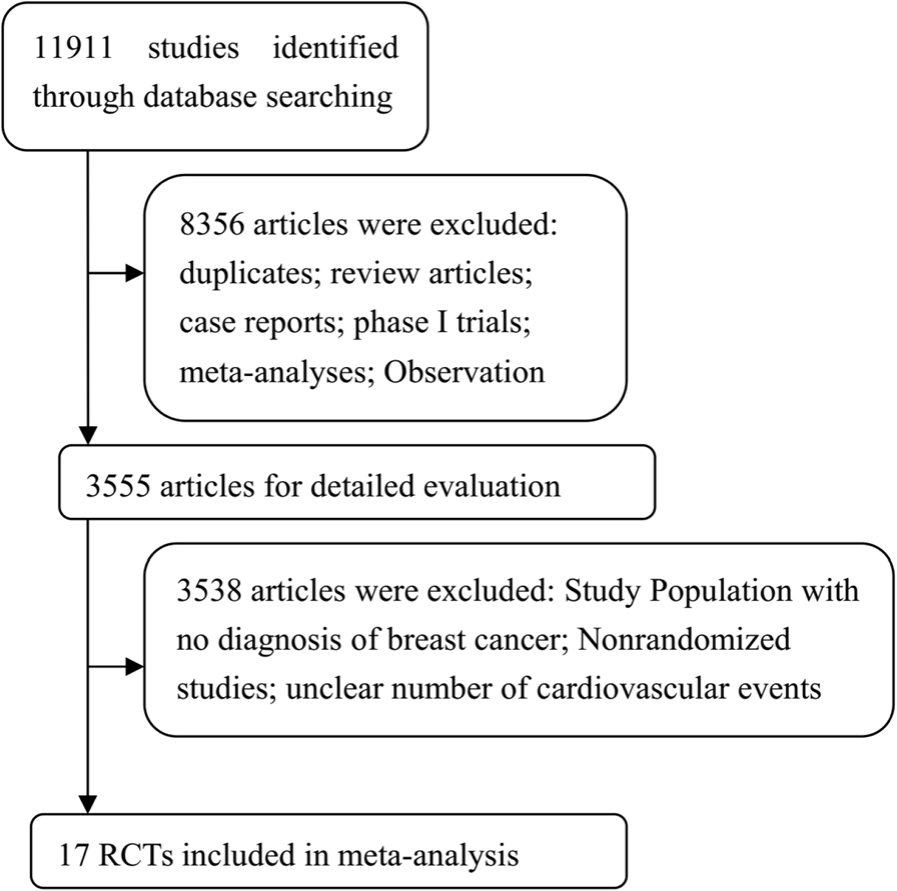
Flow chart demonstrating process of study selection

Through initial research, 11,911 potentially relevant studies were identified. After reviewing titles and abstracts, 3555 studies were performed for full-text evaluation. Ultimately, 17 studies met our inclusion criteria, and 44,411 subjects were included in our analyses [33–49]. Figure 1 outlines the selection process in detail. Of these, 15 RCTs were based in Europe [33, 35–48], 8 in North America [35, 37, 38, 43, 45–47] 6 in the Asia-Pacific region [34, 35, 38, 43, 45, 46], and several international multicenter clinical studies were included. The baseline age of subjects ranged from 29 to 96 years [35, 45]. The duration of the followup times ranged from 11.4 to 100 months [39, 40], but the majority had 30-month followup times. The quality of the 17 studies was high: five studies had Jadad scores of 5, six studies had Jadad scores of 4,and six studies had Jadad scores of 3. The detailed information is shown in Tables 1 and 2.

**Table 1:** Baseline characteristics of trials included in the meta-analysis

Author (Publication Date)	Trial phase	Tumor staging	Interventions	Age (media, years)	follow-up (media,wk)	No. of EP	Jadad score
Coombes(2007)	Phase 3 trial	Early-stage breast cancer	Exemestane (25 mg/d)		55.7 months	2320	5
Velde(2011)	Phase 3 trial	Early-stage breast cancer	Exemestane (25 mg/d)	64 (35–96)	61.2 months	9666	5
			Exemestane (25 mg/d)				
Arimidex(2008)	Phase 3 trial	Early-stage breast cancer	Anastrozole (1 mg/d)	64 (9) mean sd	100 months	3092	4
Hiroji(2013)	Phase 3 trial	Advanced-stage breast cancer	Exemestane (25 mg/d)	64 (9) mean sd	60.1 months (48.2, NR)	298	3
			Anastrozole (1 mg/d)				
Buzdar(1998)	Phase 3 trial	Advanced-stage breast cancer	Anastrozole (1 mg/d)	65.6 ± 10.9	31 months	508	3
			Anastrozole (10 mg/d)	66 ± 10.4			
Boccardo(2005)	Phase 3 trial	Early-stage breast cancer	Anastrozole (1 mg/d)	63 (38–76)	36 months	223	4
Coates(2007)	Phase 3 trial	Early-stage breast cancer	Letrozole(2.5 mg/d)		51 months	2448	5
Kaufmann(2007)	Phase 3 trial	Early-stage breast cancer	Anastrozole (1 mg/d)	60.9 mean	30.1 months	445	5
Coombes(2004)	Phase 3 trial	Early-stage breast cancer	Exemestane (25 mg/d)	64.3 ± 8.1 mean SD	30.6 months	2362	4
Paul(2013)	Phase 3 trial	Early-stage breast cancer	Exemestane (25 mg/d)	63.9	49.2 months	7576	3
			Anastrozole (1 mg/d)	64.3			
Baum(2003)	Phase 3 trial	Early-stage breast cancer	Anastrozole (1 mg/d)		33 months	3902	4
Joyce(2017)	Phase 3 trial	Advanced-stage breast cancer	Ribociclib + Letrozole(2.5 mg/d)	62.5 (37.0–82.0)		226	3
			Letrozole(2.5 mg/d)	63.0 (29.0–88.0)			
Colleoni(2017)	Phase 3 trial	Early-stage breast cancer	Continuous Letrozole(2.5 mg/d)	60 (54–67)	60 months	4828	5
			Intermittent Letrozole(2.5 mg/d)				
Tamar(2017)	Phase 2 trial	Advanced-stage breast cancer	Everolimus(10 mg/d) plus Letrozole(2.5 mg/d)	62.5 (34.6–82.0)	11.4 months	72	3
Gabe(2017)	Phase 3 trial	Advanced-stage breast cancer	Ribociclib(600 mg/d) plus Letrozole(2.5 mg/d)			674	3
			Letrozole(2.5 mg/d)				
Vivianne(2017)	Phase 3 trial	Early-stage breast cancer	Anastrozole (1 mg/d)	57.6 (51.2–64.5)	50.4 months	1660	5
			Anastrozole (1 mg/d)	57.7 (51.9–64.3)			
Smith(2017)	Phase 3 trial	Early-stage breast cancer	Letrozole(2.5 mg/d)	62 (33–96)	65 months	4111	5
			Anastrozole (1 mg/d)	62 (33–92)			

date as show with number (percentage);

*No. of EP* Number of enrolled patients; *NR* not reported

**Table 2:** Fatal or high-grade CVEs of Aromatase Inhibitors in Patients with breast cancer in our study

Author (Publication Date)	Events of CVEs							
	Hypertension	Ischaemic CV Disease	Venous thrombosis	Hypercholesterolaemia	Arrhythmia	Cardiac Failure	Peripheral Arterial Disease	Embolism
Coombes (2007)	2	4	7	0	NR	NR	NR	NR
Velde(2011)	126	76	83	NR	93	54	14	45
Arimidex(2008)	NR	NR	NR	NR	NR	NR	NR	NR
Hiroji(2013)	3	NR	NR	NR	NR	NR	NR	NR
Buzdar(1998)	NR	NR	NR	NR	NR	NR	NR	NR
Boccardo(2005)	NR	NR	NR	NR	NR	NR	NR	NR
Coates(2007)	NR	42	NR	10	NR	14	NR	25
Kaufmann(2007)	NR	NR	NR	NR	NR	NR	NR	NR
Coombes(2004)	NR	NR	NR	NR	NR	NR	NR	NR
Paul(2013)	NR	NR	NR	NR	NR	NR	NR	NR
Baum(2003)	NR	NR	NR	NR	NR	NR	NR	NR
Joyce(2017)	28	NR	NR	NR	NR	NR	NR	NR
Colleoni(2017)	1101	40	NR	NR	NR	NR	NR	38
Tamar(2017)	NR	NR	NR	1	NR	NR	NR	NR
Gabe(2017)	69	NR	NR	NR	NR	NR	NR	NR
Vivianne(2017)	NR	NR	NR	NR	NR	NR	NR	NR
Smith(2017)	45	NR	NR	3	NR	NR	NR	NR

date as show with number (percentage);

*No. of EP* Number of enrolled patients; *NR* not reported

### Overall incidence of CVEs in AIs group

**Fig. 2:**
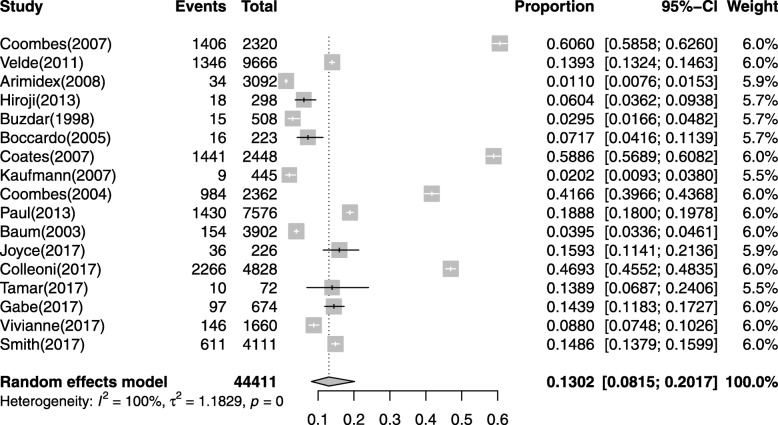
Forest plot for meta-analysis of incidence of CVEs with patients assigned AIs

A total of 59,503 subjects from the seventeen studies were available for incidence of CVEs analysis [33–49].CVEs were reported in all studies, and it ranged from 1.1 to 60.6% in AIs group. The highest incidence of CVEs was from a phase III trials of North America, which all subjects were confirmed early-stage breast cancer [47]. Based on data from each study, the calculated overall incidence of CVEs was 13.02%(95% CI: 8.15–20.17%, Fig. 2) according to the random effects model.

High-grade CVEs were reported in 10 out of 17 studies, and it ranged from 0.34 to 24.42%. The highest incidence of high-grade CVEs was from a phase III trials of Europe, which all subjects were confirmed early-stage breast cancer [42]. Based on data from each study, the calculated overall incidence of high-grade CVEs was 3.75%(95% CI: 1.66–8.24%, Additional file 1: Figure S1) according to the random effects model.

### Odds ratios of CVEs

**Fig. 3:**
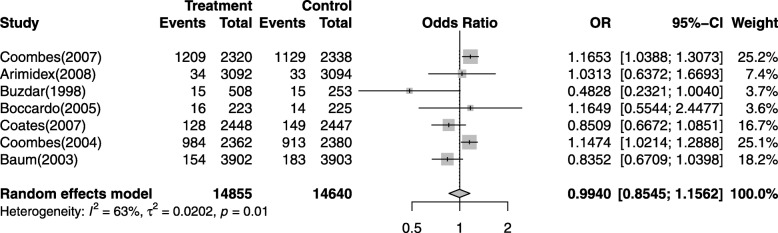
Odds ratios of AIs-associated CVEs

To evaluate the specific contribution of AIs to the development of CVEs in subjects excluding the influence of many confounding factors such as the history of course of disease, we performed the OR of CVEs between AIs and control groups. Of the 29,495 subjects from seven trials were included in OR analysis. The pooled OR for CVEs showed that treatment with AIs do not significantly increased the risk of developing CVEs in breast cancer patients with an OR of 0.9940 (95% CI: 0.8545–1.1562, *p* = 0.01, Fig. 3), according to the random effects model. The most significant difference OR was 1.1653 (95% CI: 1.0388–1.3073) occurred in a phase III study from North America [47], and high-grade CVEs occurred in ten trials [34, 35, 38, 40–43, 45–47]. Almost all of the high-grade CVEs occurred in patients treated with AIs.

### Sub-group analysis

**Fig. 4:**
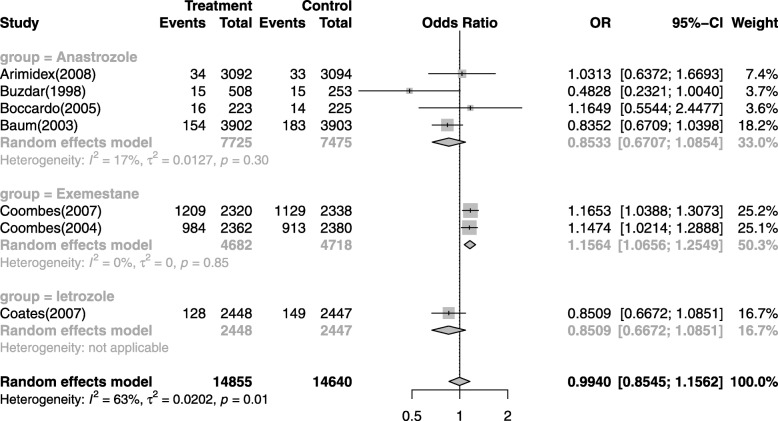
Sub-group analysis of the incidence of CVEs related to different AIs

The incidence of CVEs might be different among different tumor stages, AIs or treatment regimens. Thus, sub-group analysis was conducted according to under- lying malignancies, AIs and follow-up periods, although there is no significantly difference on the overall incidence of CVEs between patients with AIs treatment and that of non-AIs patients. There was no significant variation in the incidence of CVEs between different tumor stages (Additional file 2: Figure S2), even if the patients with advanced-stage breast cancer has often been thought of as the possible high risk factor for CVEs. Then, the incidence differences among follow-up periods were investigated and there was no significant variation of the incidence of CVEs between long time and short time follow-up periods in patients received AIs (Additional file 3: Figure S3). Additionally, we found that the incidence of CVEs related to anastrozole and letrozole was slightly higher than controls, though statistically it makes no difference. However, it is worth mentioning that the incidence of CVEs related to exemestane was higher than that of controls (OR = 1.1564, 95% CI: 1.0656–1.2549, Fig. 4), which suggested that exemestane might not be considered as the alternative AI to the breast cancer patients from the perspective of CVEs.

### Publication bias

**Fig. 5:**
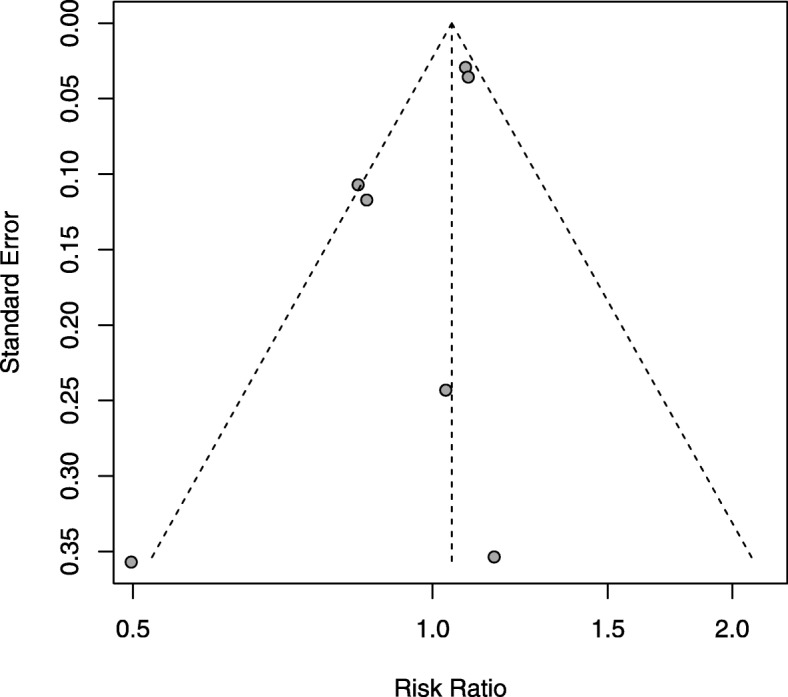
Funnel plot standard error by OR of CVEs

No evidence of publication bias was found for the OR of CVEs in our meta-analysis by a funnel plot (Fig. 5) and contour-enhanced funnel plots (Additional file 4: Figure S4 )[32].

## Discussion

It is found that CV disease and breast cancer have several overlapping risk factors, such as obesity and smoking, meanwhile cardiovascular events (CVEs) is considered as one of the major causes of death in breast cancer patients undergoing chemotherapy, and AIs may contribute to the development of CVEs [7, 50–52]. For older women, the CV disease may pose a greater mortality threat than breast cancer itself [7]. Therefore, it is vital for clinicians and patients to realize the risk of CVEs related to cancer treatment, so as to optimize the treatment strategy and actively manage these adverse events. Aromatase inhibitors (AIs) are used as standard medication therapies for most breast cancer patients [53]. However, its impact on the development of CVEs has not been sufficiently elucidated. Meanwhile, reliable data about the risk of CVEs in breast cancer patients treated with AIs is still scantly. Thus, we conducted this study to assess the risk and incidence of CVEs in breast cancer patients receiving AIs.

To our knowledge, this is the latest study to assess the CVEs risk of AIs treatment in breast cancer patients. Under the previous study, it was suggested that AIs were associated with a 19% (RR: 1.19, 95% CI: 1.07–1.34) increased risk of cardiovascular events compared with tamoxifen, and cardioprotective effects of the tamoxifen was considered to be accounted for the increased risk of cardiovascular events with AIs [51, 54]. However, it was indicated in our study that breast cancer treated with AIs do not have a significant risk of developing CVEs in comparison with the controls. However, the incidence of CVEs related to exemestane was higher than that of controls (OR = 1.1564, 95% CI: 1.0656–1.2549, Fig. 4) according to our sub-group analysis. Moreover, exemestane has a special role in the sequence of AIs during treatment of metastatic breast cancer as the drug may cause new responses following progression on non-steroidal AIs [35, 47]. Thus, it might be the other disadvantage to choose exemestane upfront based on very week data concerning the risk of CVEs. Furthermore, it was found that the the highest incidence of CVEs associated with AIs in breast cancer patients was 60.6%, and the lowest incidence of CVEs is 1.1%. The overall incidence of CVEs associated with AIs in breast cancer patients was 13.02%. In addition, the highest OR of developing CVEs with AIs versus controls in breast cancer patients was 1.1653, and the pooled OR of developing CVEs was 1.0760. More importantly, High-grade CVEs occurred in ten trials [34, 35, 38, 40–43, 45–47], and almost all of the high-grade CVEs occurred in patients treated with AIs.

Providing systemic education for breast cancer patients are important for proper management of AIs-induced CVEs. CVEs in breast cancer patients commonly including hypertension, ischemic cardiovascular disease, venous thrombosis, hypercholesterolaemia, arrhythmia, cardiac failure, peripheral arterial disease, embolism,myocardial infarction,atrial fibrillation [18–21] Usually, the incidence of CVEs is at the highest in the initial months of drug treatment and in the last stage of disease progression. On the contrary, the incidence is lower while patients responded to treatment. CVEs can lead to dose reduction and drug discontinuation in clinical treatment for breast cancer patients. Our findings clarified that the incidence of CVEs was higher in breast cancer patients receiving AIs compared to controls. In addition, the continued monitoring, effective and preventive management of CVEs are important for continued AI treatment in breast cancer patients [55]. Thus, it is very important to educate both patients and physicians about the complication and prevention.

Development of an integrated disease evaluation system which is scientific, reasonable and practical for the prediction of CVEs in AI treated patients remains a key problem for clinicians. Immediate and accurate diagnosis is essential to reduce the incidence of AIs-related CVEs. However, some major cardiovascular events, such as myocardial infarction and stroke in individuals often appear without known pre-existing cardiovascular disease, and it increases the difficulty of health management in patients with breast cancer. The prevention of CVEs and the accurate risk assessment for breast cancer patients, remains to be serious public health challenges. The developed scoring equations which use cardiovascular risk factors to predict high risk population, tend to have limited accuracy. For example, the Framingham Risk Score (FRS) which is often considered the reference standard, tend to over-estimate risk in low risk populations and under-estimate risk in high risk populations. Thus, it is recommended to incorporate more risk markers or indications, such as metabolic syndrome, plasma C-reactive protein (C-RP), coronary artery calcium (CAC), carotid intima media thickness (IMT) and the ankle brachial index (ABI) are incorporated to improve the prediction of CVEs [56]. All breast cancer patients with high risk factor for the development of AIs-induced CVEs should be carefully and cautiously cared by physicians, as well as the members of home care team.

The reasons for AIs-induced CVEs in patients with breast cancer is still confusing. The confounding factors of CVEs in breast cancer patients may from age-related condition, drug therapies or disease itself. AIs-related organ dysfunctions, such as digestive tract mucosa injury, lung and/ or renal injury also increase the incidence for CVEs [57, 58]. Older patients who suffer from physiological dysfunction and senile disease making them more susceptible to CV diseases, and physician should be aware of this while caring for these patient [59, 60]. It was shown that dietary supplements such as folic acid, vitamins B6 and vitamins B12, may reduce the rate of cardiovascular diseases, but it is uncertain if vitamin B supplementation reduces the risk of CVEs. Furthermore, recent studies with vascular diseases failed to prove the association between B-vitamin supplementation and cardiovascular diseases [13], and it needs further verification [5]. Furthermore, limited drugs such as beta blockers and angiotensin-converting enzyme inhibitors (ACEIs) were recommended to be indicated in asymptomatic patients in order to minimize the effects of chemotherapy on myocardial dysfunction, but it hasn’t been verified on patients with breast cancers [61]. Notably, practice guidelines for cancer treatments and cardiovascular toxicity was published by the the European Society of Cardiology (ESC) in 2016, so as to provide prevention and treatment strategies for CVEs [8].

Several limitations are presented in our study. Firstly, this is a meta-analysis based on previous studies and not on patient data. It is difficult to determine how the different event severity and timing of events might affect the current analyses. Moreover, there are a mix of advanced or metastatic and early breast cancer studies included in present study, and patients with metastatic breast cancer may have been exposed to a greater number of prior treatments that may also induced cardiotoxicity such as radiotherapy or anthracycline chemotherapy [62–66], which might affect cardiovascular outcomes. Thus, all of these above confounding variables including basic medication history and adjuvant therapy could not be considered in the analysis. Secondly, this meta-analysis is performed in patients with proper organ function, so the risk and sensitivity of CVEs may be higher in routine clinical practice. Thirdly, the studies were performed at various types of institutions by different researchers in this meta-analysis, and the evaluating and conclusion may be existed heterogeneous. In addition, the majority of studies have been conducted in Europe and America, which has limited the possibility to generalize the results. The limitations of the current study mean that high-quality RCTs with a large sample size are still needed to reliably evaluate the risk of AIs induced CVEs in patients with breast cancer.

Cancer-free survival rate has improved over the past 20 years for many individuals with breast cancer. However, chemotherapy associated CVEs compromised the improvement in cancer related survival [60]. As a result, there is an emerging need to obtain valuable data to optimize medical scheme for chemotherapy in patients with breast cancer by reducing the risk of CVEs. Our study showed that the incidence of CVEs in AIs group was higher compared with the controls, meanwhile almost all of high-grade CVEs occurred in patients treated with AIs. Adverse events monitoring is particularly important in reducing CVEs for AIs treatment. Optimal management and accurate diagnosis of CVEs for breast cancer patients is critical for safe medication. Besides, there is an emerging need to develop accurate, cost-effective methods to identify those individuals treated for cancer at increased risk of CVEs.

## Conclusions

In conclusion, our results suggest that patients with breast cancer treated with AIs dose not significant risk of developing CVEs in comparison with the controls. Further studies are recommended to investigate this association and the risk differences among different tumor types, AIs, ages,or treatment regimens.

## Supplementary information

**Additional file 1; Figure S1.** Forest plot for meta-analysis of incidence of High-grade CVEs with patients assigned AIs.

**Additional file 2: Figure S2.** Sub-group analysis of the incidence of CVEs between different tumor stages.

**Additional file 3: Figure S3.** Sub-group analysis of the incidence of CVEs between long time(≥24 months) and short time follow-up periods.

**Additional file 4: Figure S4.** The contour-enhanced funnel plot for standard error by OR of CVEs.

**Additional file 5: Table S1.** PRISMA 2009 checklist for systematic review.

## Data Availability

All data and materials used in this research are freely available in electronic databases (PubMed, EMBASE, Cochrane database, www.ClinicalTrials.gov) and references have been provided. The datasets used and analysed during the current study are available from the corresponding author on reasonable request. All data generated or analysed during this study are included in this published article and its supplementary information files.

## Abbreviations

ABI: Ankle brachial index
ACEIs: Angiotensin-converting enzyme inhibitors
AIs: Aromatase inhibitors
CAC: Coronary artery calcium
CI: Confidence interval
C-RP: C-reactive protein
CV: Cardiovascular
CVEs: Cardiovascular events
IMT: Intima media thickness
RCTs: Randomized control trials
