# Supplementary material for: Aromatase inhibitors and risk of cardiovascular events in breast cancer patients: a systematic review and meta-analysis
Source: BMC Pharmacol Toxicol. 2019 Oct 29;20:62. doi: 10.1186/s40360-019-0339-1 (PMC6820915; doi:10.1186/s40360-019-0339-1)
Supplement: Supplementary file 1 — Additional file 1; Figure S1. Forest plot for meta-analysis of incidence of High-grade CVEs with patients assigned AIs. [file 40360_2019_339_MOESM1_ESM.pdf]

Study	Events	Total	Proportion	95%−CI	Weight
Coombes(2007)	22	2320	0.0095	[0.0060; 0.0143]	10.6%
Velde(2011)	491	9666	0.0508	[0.0465; 0.0554]	10.9%
Hiroji(2013)	1	298	0.0034	[0.0001; 0.0186]	6.8%
Coates(2007)	113	2448	0.0462	[0.0382; 0.0552]	10.9%
Joyce(2017)	28	226	0.1239	[0.0839; 0.1741]	10.7%
Colleoni(2017)	1179	4828	0.2442	[0.2321; 0.2566]	10.9%
Tamar(2017)	1	72	0.0139	[0.0004; 0.0750]	6.8%
Gabe(2017)	69	674	0.1024	[0.0805; 0.1278]	10.8%
Vivianne(2017)	48	1660	0.0289	[0.0214; 0.0382]	10.8%
Smith(2017)	48	4111	0.0117	[0.0086; 0.0155]	10.8%
**Random effects model**		**26303**	**0.0375**	**[0.0166; 0.0824]**	**100.0%**

Heterogeneity: $I^2 = 99\%$, $\tau^2 = 1.6585$, $p = 0$

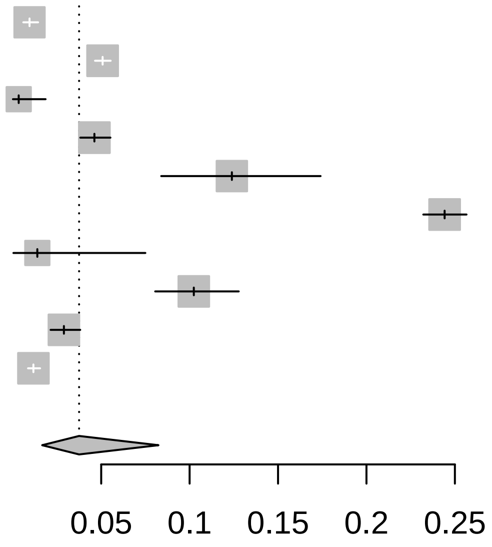

Figure S1. Forest plot for meta-analysis of incidence of High-grade CVEs with patients assigned AIs.
