# Supplementary figures and images for: Aromatase inhibitors and risk of cardiovascular events in breast cancer patients: a systematic review and meta-analysis
Source: BMC Pharmacol Toxicol. 2019 Oct 29;20:62. doi: 10.1186/s40360-019-0339-1 (PMC6820915; doi:10.1186/s40360-019-0339-1)

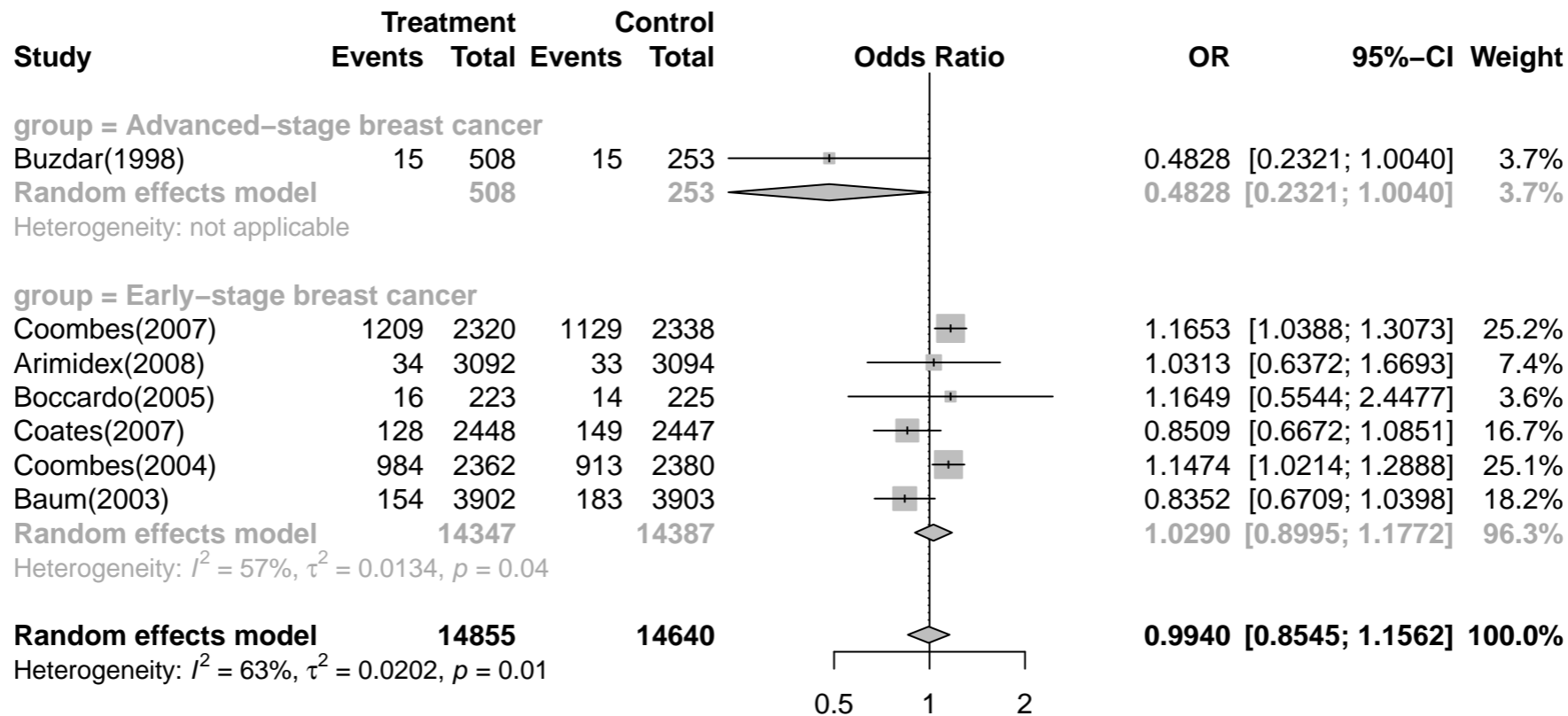

Figure S2.Sub-group analysis of the incidence of CVEs between different tumor stages.

Supplement: Supplementary file 2 — Additional file 2: Figure S2. Sub-group analysis of the incidence of CVEs between different tumor stages. [file 40360_2019_339_MOESM2_ESM.pdf]

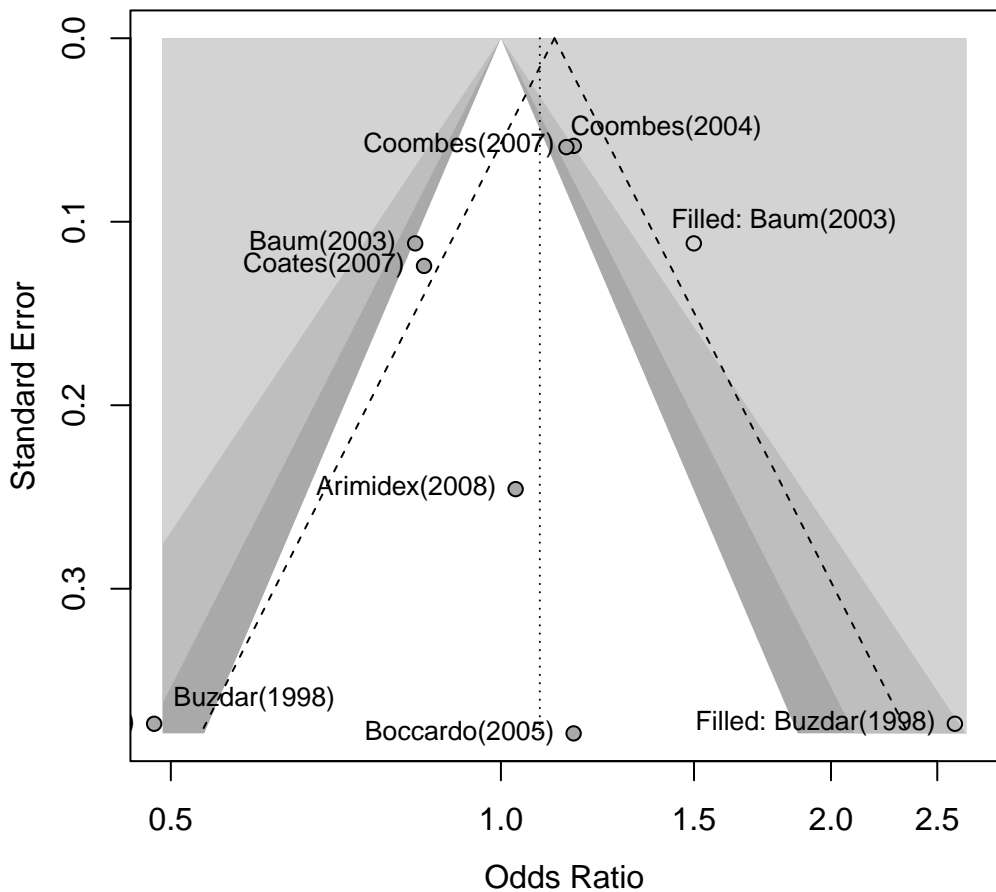

Figure S4. The contour-enhanced funnel plot for standard error by OR of CVEs.

Supplement: Supplementary file 4 — Additional file 4: Figure S4. The contour-enhanced funnel plot for standard error by OR of CVEs. [file 40360_2019_339_MOESM4_ESM.pdf]
