# Supplementary material for: Aromatase inhibitors and risk of cardiovascular events in breast cancer patients: a systematic review and meta-analysis
Source: BMC Pharmacol Toxicol. 2019 Oct 29;20:62. doi: 10.1186/s40360-019-0339-1 (PMC6820915; doi:10.1186/s40360-019-0339-1)
Supplement: Supplementary file 3 — Additional file 3: Figure S3. Sub-group analysis of the incidence of CVEs between long time(≥24 months) and short time follow-up periods. [file 40360_2019_339_MOESM3_ESM.pdf]

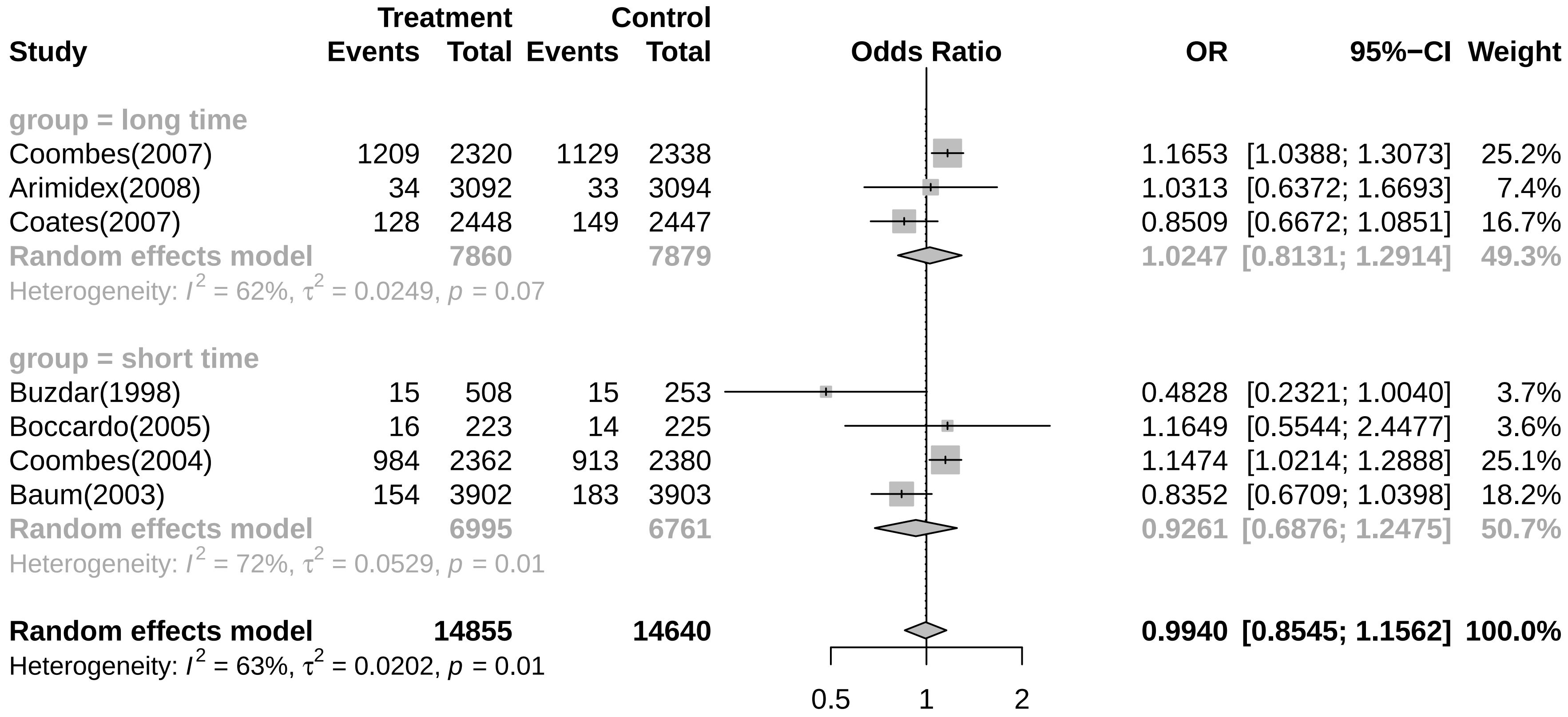

Figure S3.Sub-group analysis of the incidence of CVEs between long time and short time follow-up periods.
